# Excessive crying at 3 months of age and behavioural problems at 4 years age: a prospective cohort study

**DOI:** 10.1136/jech-2014-204568

**Published:** 2015-02-19

**Authors:** Iná S Santos, Alicia Matijasevich, Marcelo F Capilheira, Luciana Anselmi, Fernando C Barros

**Affiliations:** 1 Post-graduate Program in Epidemiology, Department of Social Medicine, Faculty of Medicine, Federal University of Pelotas, Pelotas, Rio Grande do Sul, Brazil; 2 Department of Preventive Medicine, School of Medicine, University of São Paulo, São Paulo, São Paulo, Brazil; 3 Post-graduate Program in Health and Behavior, Catholic University of Pelotas, Pelotas, Rio Grande do Sul, Brazil

**Keywords:** CHILD HEALTH, Cohort studies, EPIDEMIOLOGY, PRIMARY HEALTH CARE, PUBLIC HEALTH

## Abstract

**Background:**

Excessive crying in early infancy has been associated with behavioural problems among preschool children from high income countries but studies in low income and middle income countries are scarce.

**Methods:**

The 2004 Pelotas Birth Cohort is a population-based study planned to enrol all live births occurring in Pelotas that year and comprises 4231 children who so far have been followed up at 3, 12, 24, 48 and 72 months of age. Several familial, maternal and child characteristics were gathered in every follow-up. At the 3-month follow-up, infants whose mothers perceived them as crying more than others of the same age were classified as ‘crying babies’. Child behavioural problems were assessed through the Child Behavior Checklist (CBCL) applied to the mother at the 48-month follow-up. Crude and adjusted ORs with 95% CIs were calculated by logistic regression.

**Results:**

Prevalence of excessive crying at 3 months was 11.9% (10.9% to 13.0%). Among children with excessive crying at 3 months the proportion in the clinical range for CBCL total, internalising and externalising problems at 4 years of age was 31.2%, 12.9% and 37.5%, respectively, against 20.6%, 6.8% and 29.6%, respectively, among non-crying babies. After controlling for confounders crying babies presented increased risk of being in clinical range of CBCL total (OR=1.34; 1.03 to 1.74), internalising (OR=1.55; 1.09 to 2.21) and externalising problems (OR=1.29; 1.01 to 1.64) than infants without excessive crying.

**Conclusions:**

Excessive crying in early infancy may represent one important risk factor for developing behavioural problems in later phases of early childhood.

## Introduction

Studies have shown that psychosocial problems in childhood are associated with psychological disorders later in life and that early detection and treatment of psychosocial problems in childhood are important for prevention of psychosocial disorders in adults. Children with behaviour problems are recognised as at increased risk for problems with regulating affect, behaviour and cognition in adulthood.^1–6^

The prevalence of excessive crying during the first 3 months in representative community-based samples from high income countries has been reported to range between 14% and 29%.^7^ ^8^ There is no consensus regarding the definition of excessive crying. A frequently used definition is the excessive paroxysmal crying, that is most likely to occur about the same time every day (usually in the late afternoon or evenings) without any identifiable cause in an otherwise healthy baby aged 2 weeks to 4 months and lasting more than 3 h per day, occurring in more than 3 days in any week for 3 weeks (rule of three) that is typically known as colic.^9^ Colic affects between 9% and 12% of infants from community samples.^8^ Others give less emphasis to the amount of crying and give relevance to maternal or parental stress due to the child unresponsiveness to soothing^10^ or to the maternal perception of the intensity of crying.^11^

Negative consequences of excessive crying on maternal and child health have been described: it is associated with early weaning from breast milk,^12–14^ frequent changes of formulae,^15^ and maternal mental symptoms,^16^ besides being the most common proximal risk factor for shaken baby syndrome.^17^

The association between excessive crying in the first months of life and preschool children behaviour has been the subject of several studies in high income countries,^18–21^ whereas scarce information from low income and middle income countries is available. To explain the association between early regulatory problems (excessive crying, sleeping and feeding problems) and behavioural problems it has been suggested that the former may be early markers for similar processes of inadequate or undercontrolled behaviour in childhood.^19^ The aim of this study was to assess the prevalence of excessive crying at the age of 3 months and to test the hypothesis that excessive crying at this age is associated with behavioural problems when children reach the age of 4 years.

## Methods

A birth cohort was started in 2004 in the city of Pelotas (Southern Brazil) when all live births were included between January 1st and December 31st. Only newborns to mothers living in the urban area of Pelotas were eligible to the study. Of a total of 4263 mothers complying with this inclusion criterion, 32 (0.8%) refused to participate in the perinatal study. So far, visits have been carried out to the full cohort when children were about 3, 12, 24, 48 and 72 months of age, with follow-up rates of 95.7%, 94.0%, 93.5%, 92.0%, and 90.2%, respectively. More details of the study can be found elsewhere.^22–24^ This paper uses information collected at the perinatal study and at the 3-month and the 48-month visits.

### Exposure

Excessive crying was assessed at the 3-month visit by asking the mother whether “compared to babies of the same age, her baby cried more, less or as the same”. Infants whose mothers perceived them as crying more than others of the same age were classified as ‘crying babies’.^11^

### Outcome

Child behaviour was assessed through the application to the mother of the 4–18 year version of the Child Behavior Checklist (CBCL)^25^ when children were 4 years of age. The CBCL is designed to be self-completed (by parents), however to maximise response rates, standardisation, accuracy and to prevent embarrassment or error among less literate mothers, questions were read out by trained psychologists in the identical order and form as in the original instrument. The CBCL items are rated on a three-point Likert scale.^26^ A profile of childhood psychological problems provided scores on eight empirically derived scales: withdrawn, somatic problems, anxious/depressed, social problems, thought problems, attention problems, aggressive behaviour and rule-breaking behaviour. Data from these scales were summed to provide an overall score (total problems), and were also grouped in two broad dimensions (internalising and externalising problems). A dichotomous classification of CBCL scores comparing ‘clinical’ (positive screening) and ‘non-clinical’ (negative screening) groups was used. The clinical group was identified as those children with a t score higher than 63 points in the CBCL total, externalising or internalising scales according to the CBCL manual.^25^ In addition, CBCL scores were analysed as a continuous variable in original score units, whereby a higher score reflects greater problems. Mean CBCL scores were compared between crying and non-crying babies using Student t test. Several studies have supported the CBCL's psychometric properties, showing good reliability and validity in clinical and non-clinical populations. The CBCL was validated among Brazilian children by Bordin *et al*.^26^

### Antenatal and perinatal covariables

Information on potential confounders was gathered at the perinatal and 3-month visits. In the perinatal study, mothers were interviewed and newborns were examined during their stay at the hospital of birth. Maternal age (in complete years at delivery), skin colour (as observed by the interviewer and classified as white, black or mixed), complete years of formal education, smoking (at least 1 cigarette/day daily in at least 1 trimester of pregnancy), number of antenatal care appointments (extracted from the mother's card), type of delivery (vaginal or caesarean section), marital status (living with or without a partner), alcohol consumption during pregnancy, total family income in the month preceding parturition (subsequently divided in quintiles), and maternal heavy caffeine consumption (≥300 mg/day) in the third trimester of pregnancy were gathered at perinatal interview. Presence of maternal mood symptoms during pregnancy was defined through the positive answer to the question: “During pregnancy, did you feel depressed or have any nervous condition?”

Newborns were weighed by the hospital staff using digital paediatric scales with 10 g precision, calibrated weekly to standard weights and birth length was measured by research team using a locally made infantometer. Gestational age was calculated using the first day of the last normal menstrual period or estimated by obstetric ultrasound obtained before 20 weeks of gestation or was estimated from physical and neurological assessment of the newborn (Dubowitz *et al*’s^27^ method). Information on preterm birth (<37 weeks of gestational age), firstborn (yes or not), Apgar's score at the fifth minute of life (<7 and ≥7), and type of hospital admission for the newborn (‘staying with the mother’ or “neonatal intermediate or intensive care”) was collected.

### Three-month postpartum covariables

At 3 months post partum, maternal mental health was evaluated through the application of the Self-Reporting Questionnaire (SRQ-20).^28^ ^29^ A validation study in Brazil defined the cut-off ≥8 to identify women in higher risk of common mental disorders.^30^ Information on maternal heavy caffeine consumption (≥300 mg/day), breastfeeding pattern (exclusive, predominant, partial or weaning) was collected. Bed sharing (mother and child sharing the same surface at night to sleep),^31^ and use of pacifier was also gathered at 3 months of age.

### Statistical analyses

Only infants from singleton pregnancies were included in the analyses. Prevalence rates of excessive crying with 95% CIs (PR; 95% CI) were calculated for every maternal and child characteristic. The association between excessive crying and CBCL scores was assessed through logistic regression. A hierarchical model was used to control for confounders. Variables associated with excessive crying and positive CBCL score at a p level ≤0.20 were kept in the regression model as confounders. In the first step (model 1) the crude effect of excessive crying was measured. In the second step (model 2) the association between excessive crying and behavioural problems was adjusted for maternal antenatal (antenatal care, smoking in pregnancy, caffeine intake in third trimester and maternal mood symptoms in pregnancy) and perinatal variables (family income, maternal age and maternal education), as well as for the newborn characteristics (Apgar at 5th minute and firstborn child). In the third step (model 3), potential confounders from the 3-month follow-up were added to variables of model 2 (maternal SRQ-20, breastfeeding pattern, and bed sharing). ORs with 95% CIs were obtained with Stata V.12.0.

### Ethics

The perinatal study and all cohort follow-ups had the research protocol approved by the Medical Research Ethics Committee of the Federal University of Pelotas, affiliated to the Brazilian National Commission for Research Ethics (CONEP). Mothers were requested to fill a written consent for participation in the study.

## Results

A total of 3674 mothers replied to the question on excessive crying and to the CBCL questionnaire and were included in the analyses. Prevalence of excessive crying was 11.9% (95% CI 10.9% to 13.0%). Table 1 presents the sample distribution and prevalence of excessive crying according to independent variables. Among the maternal antenatal characteristics, excessive crying was negatively associated with younger age and higher level of formal education. Maternal mood symptoms during pregnancy and positive SRQ-20 at 3 months post partum were strongly associated with excessive crying.

**Table 1:** Sample distribution and excessive crying prevalence rates with 95% CIs according to maternal and child characteristics

	N (%)	Prevalence of excessive crying	95% CI
*Antenatal and perinatal maternal and child characteristics*			
Family income (n=3578)		p=0.070*	
1 (poorest)	707 (19.8)	13.1	(10.7 to 15.7)
2	716 (20.0)	12.3	(9.9 to 14.7)
3	718 (20.1)	13.1	(10.6 to 15.6)
4	752 (21.0)	10.8	(8.6 to 13.0)
5 (richest)	685 (19.1)	10.4	(8.1 to 12.7)
Maternal age (n=3576)		p=0.020*	
<19	677 (18.9)	8.3	(6.2 to 10.4)
20–34	2405 (67.3)	12.9	(11.5 to 14.2)
≥35	494 (13.8)	12.4	(9.4 to 15.3)
Maternal skin colour (n=3578)		p=0.574†	
White	2613 (73.0)	11.8	(10.5 to 13.0)
Black and mixed	965 (27.0)	12.4	(10.4 to 14.7)
Maternal education (years of study; n=3578)		p=0.002*	
0–4	534 (15.1)	16.5	(13.3 to 19.6)
5–8	1475 (41.6)	12.3	(10.6 to 13.9)
9–11	1189 (33.5)	10.0	(8.3 to 11.7)
≥12	347 (9.8)	10.7	(7.4 to 13.9)
Live with partner (n=3578)		p=0.266†	
No	560 (15.7)	10.5	(8.0 to 13.1)
Yes	3018 (84.4)	12.2	(11.0 to 13.4)
Number of antenatal care appointments (n=3523)		p=0.139†	
0–4	363 (10.6)	14.9	(11.4 to 19.0)
5–10	2380 (69.3)	11.9	(10.7 to 13.3)
≥11	691 (20.1)	10.7	(8.5 to 13.3)
Smoking (n=3578)		p=0.125†	
No	2608 (72.9)	11.4	(10.2 to 12.6)
Yes	970 (27.1)	13.3	(11.2 to 15.4)
Alcohol in pregnancy (n=3578)		p=0.682†	
No	3456 (96.6)	11.9	(10.8 to 13.0)
Yes	122 (3.4)	13.1	(7.0 to 19.2)
Type of delivery (n=3578)		p=0.711†	
Vaginal	1974 (55.2)	11.8	(10.3 to 13.2)
C-section	1604 (44.8)	12.2	(10.6 to 13.8)
Caffeine intake 3rd trimester (n=3310)		p=0.150†	
<300 mg/day	2724 (82.3)	11.5	(10.3 to 12.7)
≥300 mg/day	586 (17.7)	13.7	(10.9 to 16.4)
Maternal mood symptoms in pregnancy (n=3577)		p<0.001†	
No	2691 (75.2)	10.7	(9.5 to 11.9)
Yes	886 (24.8)	15.7	(13.3 to 18.1)
Sex of the newborn (n=3578)		p=0.219†	
Male	1861 (52.0)	12.6	11.1 to 14.1
Female	1717 (48.0)	11.2	(9.7 to 12.7)
Gestational age at birth (n=3574)		p=0.752*	
<34	85 (2.4)	15.3	7.5 to 23.1
34–36	372 (10.4)	12.1	8.8 to 15.4
37–41	2884 (80.7)	11.8	10.6 to 12.9
≥42	233 (6.5)	12.9	8.5 to 17.2
Low birth weight (n=3577)		p=0.603†	
No	3290 (92.0)	11.9	10.7 to 13.0
Yes	287 (8.0)	12.9	9.0 to 16.8
Neonatal intensive care (n=3569)		p=0.234†	
No	3393 (95.1)	11.8	10.7 to 12.9
Yes	176 (4.9)	14.8	9.5 to 20.1
Apgar 5th minute (n=3560)		p=0.057†	
<7	55 (1.5)	3.6	0.0 to 8.7
≥7	3505 (98.5)	12.0	10.9 to 13.1
Firstborn (n=3577)		p<0.001†	
No	2006 (56.1)	14.0	(12.5 to 15.5)
Yes	1571 (43.9)	9.3	(7.9 to 10.7)
*Maternal and infant characteristics at 3 months post partum*			
Caffeine intake (n=3316)		p=0.718†	
<300 mg/day	2817 (85.0)	11.9	10.7 to 13.1
≥300 mg/day	499 (15.0)	12.4	9.5 to 15.3
SRQ≥8 (n=3569)		p<0.001†	
No	2649 (74.2)	9.3	(8.2 to 10.4)
Yes	920 (25.8)	19.5	(16.9 to 22.0)
Breastfeeding (n=3578)		p=0.154*	
Weaned	945 (26.4)	12.5	10.4 to 14.6
Partial	990 (27.7)	13.1	11.0 to 15.2
Predominant	670 (18.7)	12.2	9.8 to 14.7
Exclusive	973 (27.2)	10.0	8.0 to 11.9
Bed sharing (n=3578)		p=0.001†	
No	1915 (53.5)	10.3	8.9 to 11.6
Yes	1663 (46.5)	13.8	12.2 to 15.5
Use of pacifier (n=3578)		p=0.557†	
No	1220 (34.1)	12.4	10.5 to 14.2
Yes	2358 (65.9)	11.7	10.4 to 13.0

*Test for trend.

†χ^2^ test.

SRQ, Self-Reporting Questionnaire.

None of the newborn characteristics were associated with excessive crying except of not being a firstborn (table 1). At 3 months, infants who shared the bed with their mothers to sleep during the night presented higher prevalence of excessive crying.

Among children with excessive crying at 3 months the proportion in the clinical range for CBCL total, internalising and externalising problems at 4 years of age was 31.2%, 12.9% and 37.5%, respectively. Among non-crying babies CBCL total, internalising and externalising problems were 20.6%, 6.8% and 29.6%, respectively. Table 2 shows the means, SDs and ranges for CBCL total, internalising and externalising problems at the age of 4 years, according to the exposure status at 3 months old.

**Table 2:** Mean, SD and range of CBCL total, internalising and externalising scales at 4 years of age according to excessive crying at the age of 3 months

CBCL scales	Crying babies			Non-crying babies			p Value*
	Mean	SD	Range	Mean	SD	Range	
Total CBCL	39.5	18.7	1–135	34.0	15.8	5–132	<0.001
Internalising problems	7.6	5.7	0–43	6.1	4.4	0–32	<0.001
Externalising problems	17.0	7.9	1–46	15.3	7.0	1–52	<0.001

*Student t test.

CBCL, Child Behavior Checklist.

In the crude analysis crying babies had higher risk of being in the clinical range for CBCL total, internalising and externalising problems than babies who did not cry a lot (table 3). In the full-adjusted model allowing for antenatal, perinatal and 3-month variables, the magnitude of the association between excessive crying and behavioural problems decreased. However, the association remained statistically significant and babies with excessive crying showed higher risk of being in the clinical range of CBCL total (OR=1.34; 95% CI 1.03 to 1.74), internalising (OR=1.55; 95% CI 1.09 to 2.21) and externalising problems (OR=1.29; 95% CI 1.01 to 1.64) than infants without excessive crying (table 3).

**Table 3:** Crude and adjusted ORs and 95% CIs for CBCL clinical scores (total, internalising and externalising syndromes) at 48 months of age

	Models	CBCL total		Internalising syndrome		Externalising syndrome	
		OR (95% CI)	p Value	OR (95% CI)	p Value	OR (95% CI)	p Value
Excessive crying	1	1.75 (1.40 to 2.18)	<0.001	2.04 (1.49 to 2.80)	<0.001	1.50 (1.21 to 1.85)	<0.001
Model 1+antenatal and perinatal variables*	2	1.57 (1.22 to 2.02)	<0.001	1.77 (1.26 to 2.50)	0.001	1.43 (1.12 to 1.81)	0.003
Model 2+3-month variables†	3	1.34 (1.03 to 1.74)	0.028	1.55 (1.09 to 2.21)	0.015	1.29 (1.01 to 1.64)	0.042

*Adjusted for family income, maternal age, maternal education, antenatal care, smoking in pregnancy, caffeine intake in third trimester, maternal mood symptoms in pregnancy, Apgar at fifth minute, and firstborn child. †Adjusted for variables of the second model+maternal SRQ-20, breastfeeding pattern, and bed sharing at 3 months.

CBCL, Child Behavior Checklist; SRQ, Self-Reporting Questionnaire.

## Discussion

This study showed that excessive crying is a prevalent problem and that mothers of crying babies at the age of 3 months reported more problem behaviours when their children reach 4 years of age than mothers of non-crying babies. The reported PR of excessive crying was lower than the described in studies from high income countries. Genetic predisposition, care giving practice or soothing techniques, as well as ethnic differences in maternal perceptions of crying might be among the explanations for these differences. Overall PR may also vary strongly according to the operational definition of excessive crying employed, as well as according to the way the information is collected (retrospective or prospective parental report, diaries or taping registration). In a population-based cross-sectional study involving 3345 Dutch infants at 1–6 months of age, 10 definitions that had been used in previous studies were compared, showing that the overall PR of excessive crying may vary eightfold depending on the definition used.^8^ In a population-based study conducted with 1826 infants aged 2–3 months in Amsterdam, overall prevalence of ‘crying for three or more hours/day’ in the previous week, ‘crying a lot’, and ‘difficult to comfort’ were 7.6%, 14.0%, and 10.3%, respectively.^11^ One-fifth (20.3%) of the mothers answered affirmatively to at least one criterion, whereas 14% met all three criteria.^11^ As seen by the 95% CI the PR under the criterion ‘baby cried a lot’ (14.0%; 12.4% to 15.6%)^11^ was similar to the observed in the current study.

As perceived by their mothers, crying babies presented a higher prevalence of behavioural problems for both domains of the CBCL at 4 years of age in comparison to non-crying babies. Externalising syndromes were more frequently reported than internalising problems probably because externalising disorders are more easily observable than internal states.^32^ Perception of the child behaviour as a problem may also be influenced by the presence of maternal depression symptoms and stress.^32–34^ It is also possible that the lower rate of internalising problems reported is due to the fact that tears and fears may simply be regarded by mothers as common and age-appropriate emotions for 4-year-olds.

The increased risk for problem behaviour observed among crying babies is in agreement with findings from high income countries, and may have important implications for maternal counselling on childcare. A meta-analysis including 22 longitudinal studies to test the association between infant regulatory problems (excessive crying, sleeping and feeding problems) and long-term behavioural outcomes in childhood showed that the presence of previous regulatory problems was associated with behavioural problems in childhood and that crying problems led to the highest effect sizes.^19^

Although excessive crying is usually a benign and self-limiting neurodevelopmental phenomenon, with no need of additional investigations for a child with no signs of illness on a thorough history and examination,^35^ ^36^ early detection and management of babies who cry a lot may play a role in the primary prevention of behavioural problems in childhood and in adult life. Several methods have been tried to prevent or manage crying babies in the first months of life. Studies comparing parenting style showed that proximal care (prolonged holding, frequent breast feeding, rapid response to infant frets and cries, and bed sharing with infants at night) or even moderate levels of physical contact from birth result in infants with less crying problems than more structured approaches of caring.^37^ ^38^

At the opposite, St James-Roberts *et al*^39^ compared the effectiveness of two interventions introduced from birth (supplementary carrying, increased parental responsiveness) with a control group in reducing the amounts of crying in general community infants at 2, 6, and 12 weeks age. The authors found no differences in amounts of crying and fussing between the three groups of infants.^39^ Additionally, a comprehensive literature review on the effect of behavioural interventions to reduce parental distress caused by excessive infant crying in the first 6 months of life showed that despite substantial investment in recent years in implementation and evaluation of first wave behavioural interventions for infant sleep in the first 6 months, these strategies have not shown to decrease infant crying, prevent sleep and behavioural problems in later childhood, or protect against postnatal depression, besides being subject to important methodological constraints.^40^

The strength of this study is the large number of participants with a high rate of follow-up and the population-based design, besides the fact that the predictor variable was assessed in the past when infants were 3 months old, which avoids selective recall bias. This study has several limitations. First, the outcome had to rely on mother-reports of problem behaviour, as the children were too young for self-reports or assessments by teachers or other informants. Second, is the lack of objective, non-maternal evidence that infants reported as crying babies actually cried more than others of the same age. Data on parental behaviour and infant crying collected by parents using behaviour diaries as compared with audio recordings and researcher observations showed that diaries are the leading method for studies of infant crying.^37^ ^41^ ^42^ However, audiotape recordings are too expensive for use in large samples and prospective diaries in general are not completed in low socioeconomic groups giving place for misclassification bias. Third, similar crying may not be interpreted in the same way by all mothers,^43^ and babies with excessive crying are not a homogenous group, but rather are part of subgroups that differ in their natural course or aetiology.^8^ ^35^ ^44^ And fourth, the effect of comorbidity of other regulatory problems in the children (problems with feeding and sleeping) that can contribute to the occurrence of behavioural problems later in childhood,^19^ was not assessed at the study.

## Conclusion

This study showed that while most babies passed through the crying period without long-term effect, a small subgroup of crying babies will screen positively to behavioural problems in later childhood. Considerable gaps and controversies still remain in regard to the effectiveness of available interventions, and further research into prevention and early intervention is required.^45^ ^46^

Meanwhile, simple general rules such as feeding a hungry baby, changing wet diapers, and comforting a baby who is cold and crying as a result of these, as well as parental attention, including eye contact, talking, touching, rocking, walking and playing, may be effective in some infants and is never harmful.^17^ ^38^

**What is already known on this subject**

- Studies with children from high income countries show that excessive crying is a precursor of many problems that emerge in later phases of childhood but studies on this subject from low income and middle income countries are scarce at the literature.

**What this study adds**

- After controlling for several confounders, mothers of crying babies at the age of 3 months belonging to a large population-based birth cohort study in a middle income country reported more internalising and externalising problem behaviours when their children reached 4 years of age than mothers of non-crying babies.
